# The Effect of Process Parameters on the Temperature and Stress Fields in Directed Energy Deposition Inconel 690 Alloy

**DOI:** 10.3390/ma17061338

**Published:** 2024-03-14

**Authors:** Chen Liu, Yu Zhan, Hongjian Zhao, Shuo Shang, Changsheng Liu

**Affiliations:** 1 Key Laboratory for Anisotropy and Texture of Materials (Ministry of Education), School of Materials Science and Engineering, Northeastern University, Shenyang 110819, China; shangs@mail.neu.edu.cn; 2 College of Sciences, Northeastern University, Shenyang 110819, China; zhanyu@mail.neu.edu.cn (Y.Z.); 2100308@stu.neu.edu.cn (H.Z.)

**Keywords:** additive manufacturing, directed energy deposition, Inconel 690, residual stress, finite element simulation

## Abstract

Additive manufacturing (AM) technology has the advantages of designability, short process times, high flexibility, etc., making it especially suitable for manufacturing complex high-performance components for high-end industrial systems. However, the intensive temperature gradients caused by the rapid heating and cooling processes of AM can generate high levels of residual stresses, which directly affect the precision and serviceability of the components. Taking Inconel 690 alloy, which is widely used in nuclear power plants, as the research object, a thermo-coupled mechanical model of temperature field and residual stress field of directed energy deposition (DED) of Inconel 690 was established based on ABAQUS 2019 finite element software to study the influence of process parameters on the temperature history and the distribution of residual stresses in the DED process. The experimental results show that the peak temperature of each layer in the fabrication process increases with the increase in laser power and preheating temperature, and decreases with the increase in scanning speed and interlayer dwell time. Substrate preheating only has a large effect on the peak temperature of the first four layers. Residual stresses are mainly concentrated in the upper and middle parts, the bottom of the substrate, and the sides combined with the substrate, and the residual stresses increase with the increasing laser power and decrease with the increasing interlayer dwell time. Decreasing laser power, longer dwell time, higher preheating temperature, and appropriate scanning speed are beneficial for the reduction in residual stresses in Inconel 690 components. This research has important significance for the process design and residual stress modulation in the additive manufacturing of Inconel 690 alloy.

## 1. Introduction

Inconel 690 alloy is a high-chromium nickel-based corrosion-resistant alloy. It has excellent resistance to stress corrosion cracking, high strength, and good weldability. It is widely used in the manufacture of key components of pressurized water reactor (PWR) nuclear power plants, such as reactor pressure vessels, steam generators, and voltage regulators [1]. It is well known that the traditional nuclear equipment manufacturing process is highly labor- and capital-intensive. Taking steam generators as an example, traditional manufacturing mainly involves forging, machining, welding, and post-weld heat treatment. The manufacturing cycle of a steam generator is typically 2–3 years. The stress-relieving process alone takes 6 months [2]. As a result, the product has a long lead time, high cost, and many control variables, which ultimately makes it difficult to achieve the rapid manufacturing of complex structural components.

In recent years, several additive manufacturing (AM) technologies have been developed, which have the potential to revolutionize the design and manufacture of parts. Additive manufacturing has already been successfully applied in aerospace, biomedical, and other fields [3,4,5]. The U.S. Nuclear Regulatory Commission (NRC) has proposed additive manufacturing as an innovative manufacturing process to reduce the time required to deploy advanced materials and fabricate them. Several studies have been conducted in this area. Zhong et al. [6] deposited SS316L nuclear nozzles directly onto a large-curvature pressurized water reactor (PWR) main pipes using wire arc additive manufacturing (WAAM) and cold metal transfer (CMT) processes. Kang et al. [7] used directed energy deposition (DED) to fabricate the body, bonnet, and cage components of a three-inch nuclear safety class 1 valve. Westinghouse has fabricated 316L stainless steel, Inconel 718, and Zr alloys as nuclear reactor components using powder bed fusion (PBF) [8]. These technologies are not yet widely used for manufacturing nuclear power plant (NPP) components, but have the potential to dramatically reduce manufacturing costs and time, combine multiple system and assembly components into a single part, and improve safety and reliability. The advantages of DED include the ability to produce high-density metal parts with excellent metallurgical properties at a reasonable production rate, as well as the ability to repair and remanufacture structural components; however, DED also exhibits the cumulative effect of complex heating and cooling cycles during powder deposition, leading to high residual stresses in the deposited parts, which can further lead to cracks inside the material [9]. Residual stresses are detrimental to the fatigue performance of the component and affect its dimensional stability and machining accuracy. Non-destructive testing techniques such as X-ray diffraction [10], neutron diffraction [11], and laser ultrasonic methods [12] have been used to detect residual stresses in additively manufactured parts. Additionally, destructive methods such as drilling [13], contouring [14], and nanoindentation [15] are applied to detect residual stresses. In laser additive manufacturing, the temperature and stress changes during processing are rapid, and the stress changes in the forming process cannot be accurately measured in real time using traditional testing methods.

In comparison, finite element modeling has become an alternative and economical method to understand the underlying thermo-mechanical behavior and predict residual stresses in parts. Sun et al. [16] used the finite element method to investigate the temperature and stress fields of five typical rectangular structures and concluded that the equivalent residual stresses and the maximum principal residual stresses of the S-type structure can be minimized. Zhao et al. [17] investigated the residual stresses of additively manufactured Ti-6Al 4V/Inconel 718 gradient materials, and found that the laser power is positively correlated with the structural residual stress, and the change in residual stress at the contact surface is relatively flat when the components of the two gradient materials are close to each other. Mukherjee et al. [18] used coupled thermal, fluidic, and mechanical modeling to investigate the stress and strain evolution of Inconel 718 and Ti-6Al-4V and its dependence on key process parameters such as heat input and layer thickness, and showed that residual stresses can be significantly reduced by reducing the layer thickness during the additive manufacturing process. A reduction in residual stresses of approximately 20% can be achieved by doubling the heat input. Erik R. Denlinger et al. [19] introduced the stress relaxation effect into the DED finite element model to study the residual stress and deformation of Ti-6Al-4V and Inconel 625 with different interlayer dwell times; the results showed that in the deposition process, increasing the dwell time reduces the deformation and residual stress in Inconel 625. The opposite is true for Ti-6Al-4V, where the decrease in dwell time leads to a significant reduction in the level of residual stress and deformation. Vastola et al. [20] used finite element modeling to investigate the effects of beam size, beam power density, beam scanning speed, and chamber bed temperature on the residual stresses and deformation of Ti-6Al-4V alloys melted by the e-beam melting method. The results show that for each 50 °C increase in the preheating temperature, the residual stress decreases by about 20%. McCracken et al. [21] used SysWeld to simulate narrow bevel welding of Inconel 690 and concluded that DDC occurs predominantly in the region of multiple reheat cycles and high strain accumulation. Yin et al. [22] investigated the variation in temperature distribution and residual stress distribution in single V-shaped butt welds and found that the residual stress increased with the number of layers, and the highest residual stress reached 340 MPa in the second layer. Wu et al. [23] simulated the temperature and stress fields of the Inconel 690/321 welding process in a modular small reactor. The results show that the circumferential residual tensile stress is concentrated in the last weld and the immediate sides, with a maximum tensile stress of 406 MPa, while the residual stress away from the weld base material is almost 0 MPa.

In summary, the process parameters have a significant effect on residual stresses in additive manufacturing, and the laws of influence are varied for different materials. There is a limited amount of research on residual stresses in Inconel 690 alloys using DED. Most of the available investigations are single-pass or multi-pass welding simulations, with relatively simple thermal histories. There are limited studies on complex shapes and multi-pass, multi-layer overlay welding or additive manufacturing, where complex changes in thermal history can lead to intricate stress distributions. Therefore, this paper presents a thermo-mechanical model of DED-fabricated Inconel 690 alloy to predict the temperature and stress fields using the finite element method. This paper investigates the influence of process parameters such as power, speed, interlayer dwell time, and substrate preheating temperature on the temperature and stress fields of Inconel 690. The results of this study provide an informative basis for the understanding and rationalization of the control of residual stresses in additively manufactured Inconel 690.

## 2. FEM Model

### 2.1. Thermal Analysis

**Figure 1:**
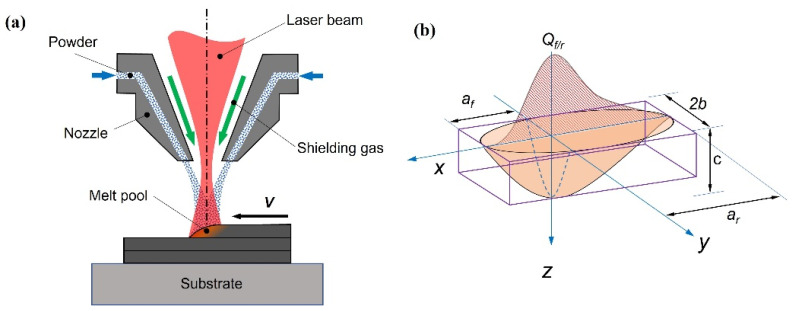
(**a**) Schematic diagram of DED process; (**b**) Goldak’s double ellipsoid heat source model for the DED process.

A thermo-mechanical finite element model of directed energy deposition (DED) was constructed using ABAQUS 2019 commercial finite element software. Figure 1 shows a schematic diagram of the metal powder DED process. When a laser irradiates the surface of the substrate, the surface is rapidly melted and a molten pool is formed. A powder nozzle delivers the metal powder directly into the molten pool, where it rapidly melts and solidifies, forming a metallic cladding layer fused to the substrate.

The calculation of residual stresses and structural deformation relies heavily on a reasonable heat source model. In this study, we used the Goldak double ellipsoid heat source model to describe the energy absorption mechanism of a laser beam on the substrate. A visual representation of the Goldak double ellipsoid heat source model is shown in Figure 1b. The Goldak double ellipsoid heat source model is commonly used for thermal simulations of moving heat sources in additive manufacturing [19]. The energy density distribution of the model is defined by the following equation:

(1) $$Q(x,y,z)=\left\{\begin{array}{l} \frac{6\sqrt{3}f_{f}AP}{a_{f}bc\pi \sqrt{\pi }}\exp\left(-\frac{3x^{2}}{a_{f}^{2}}-\frac{3y^{2}}{b^{2}}-\frac{3z^{2}}{c^{2}}\right),(x⩾0)\\ \frac{6\sqrt{3}f_{r}AP}{a_{r}bc\pi \sqrt{\pi }}\exp\left(-\frac{3x^{2}}{a_{r}^{2}}-\frac{3y^{2}}{b^{2}}-\frac{3z^{2}}{c^{2}}\right),(x<0) \end{array}\right.$$

where *a_r_* and *a_f_* are the front and rear radius of the double ellipsoid on the *x*-axis, respectively, *b* is the length of the radius of the double ellipsoid on the *y*-axis, *c* is the depth of laser penetration, *A* is the absorption rate of the laser light by the metal powder, *P* is the laser power, and *f_f_* and *f_r_* are dimensionless fractions of the heat in the front and the rear halves of the laser, respectively.

During the DED process, the temperature changes rapidly and dramatically. It is a nonlinear transient heat transfer problem. Its governing equation can be expressed as:

(2) $$C_{p}\rho \frac{dT}{dt}=\frac{\partial }{\partial x}\left(k\frac{dT}{dt}\right)+\frac{\partial }{\partial y}\left(k\frac{dT}{dt}\right)+\frac{\partial }{\partial z}\left(k\frac{dT}{dt}\right)+Q$$

where *ρ* is the density, *C_p_* is the specific heat capacity, *T* is the temperature, (*x*, *y*, *z*) are the coordinates, *t* is time, *k* is the thermal conductivity, and *Q* is the heat flux, which is the amount of heat per unit volume that is introduced into the block from an external source.

### 2.2. Mechanical Analysis

The results of temperature field distribution are imported into the mechanical analysis model as the load of stress analysis to realize the coupled calculation of temperature field and stress field. The stress–strain equation is

(3) $$\sigma_{ij}=C\cdot \varepsilon^{e}$$

where *σ_ij_* is the stress tensor, *C* is the fourth-order material stiffness tensor, and *ε^e^* is the elastic strain tensor.

The strains in the structure are defined as follows:

(4) $$\varepsilon^{t}=\varepsilon^{e}+\varepsilon^{p}+\varepsilon^{th}$$

where *ε^t^* is the total strain vector, *ε^e^* is the elastic strain, *ε^p^* is the plastic strain, and *ε^th^* is the thermal strain. Thermal strain is defined as:

(5) $$\varepsilon^{th}=\alpha \left(T-T_{ref}\right)$$

where *α* is the temperature dependent coefficient of thermal expansion and *T_ref_* is the ambient temperature.

Tensile tests of the Inconel 690 material used in the experiments of this study showed extensive plastic deformation and limited work hardening [24]. The mechanical analysis used the ideal plastic material model, which is a commonly used model in the literature [25,26].

### 2.3. Boundary Conditions

Consider the energy loss at the surface of the part due to thermal radiation and convection, expressed as follows:

(6) $$T\left(x,y,z,0\right)=T_{0}$$

(7) $$k\frac{\partial T}{\partial n}-q+h_{c}\left(T-T_{0}\right)+\sigma_{b}\varepsilon_{b}\left(T^{4}-T_{0}^{4}\right)=0,\left(x,y,z\right)\in S_{n}$$

where *T*_0_ is the ambient temperature, *S_n_* is the magnitude of the normal vector on the model surface, *h_c_* is the natural convective heat transfer coefficient, *σ_b_* is the Stefan–Boltzmann constant, and *ε_b_* is the radiative emittance.

### 2.4. Mesh Modeling and Material Properties

**Figure 2:**
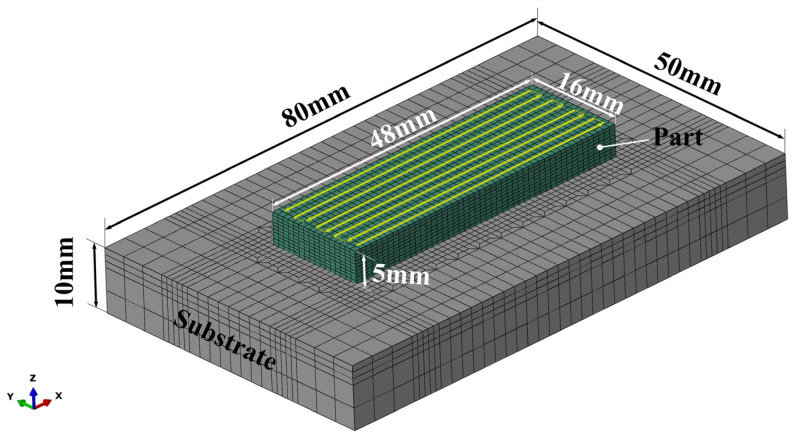
Finite element model of the substrate and part geometry for the LDM simulation.

The accuracy of numerical simulation predictions relies heavily on the precision of the mesh and the temperature-dependent material property data. To capture steep temperature gradients at specific time steps, a fine mesh was generated in and around the deposition region of the part. The modeling was performed using ABAQUS 2019 commercial software, and the finite element model comprised 16,640 cells and 19,472 nodes, as illustrated in Figure 2. For the analysis of the temperature field, both the substrate and the deposition layer were assessed using DC3D8 heat transfer cells. For the analysis of the stress field, C3D8 cells were used. The DED process involves dynamically injecting metal powder into the molten pool, and the numerical analysis model adopts the “progressive activation element” method to simulate the dynamic generation of the deposition layer [27].

In the FE model, a single 48 × 16 × 10 mm block was deposited on the 80 × 50 × 10 mm substrate. There were 10 layers in the FE model. In this study, Inconel 690 alloy had a solid phase temperature of 1140 °C, a liquid phase temperature of 1390 °C, and a latent heat of 246.2 J/g. Considering the simplified model, the material density was taken as 8.16 g/cm^3^, independent of temperature. Boundary conditions for convective and radiative heat transfer were applied to all free surfaces of the model. The forced convective heat transfer coefficient was 40 W/(m^2^·°C) and the emissivity parameter was 0.4. The energy absorption coefficient A was 0.40 and the ambient temperature was maintained at 25 °C throughout the DED process. The substrate was also Inconel 690 with no constraints applied. Figure 2 shows the deposition strategy used in the simulation. Extensive code was written in Python to simulate the scan path of the laser heat source and the deposition process of the Inconel 690 alloy. The numerical simulations were carried out on a computer equipped with an Intel i7 8700 hexa-core processor (3.20 GHz) and 16 GB of RAM.

Table 1 lists the temperature-dependent thermophysical properties of Inconel 690—thermal diffusivity, thermal conductivity, isobaric heat capacity, Young’s modulus, Poisson’s ratio, and yield strength—which were considered in the thermomechanical simulations [28].

## 3. Results and Discussion

### 3.1. Temperature Field

**Figure 3:**
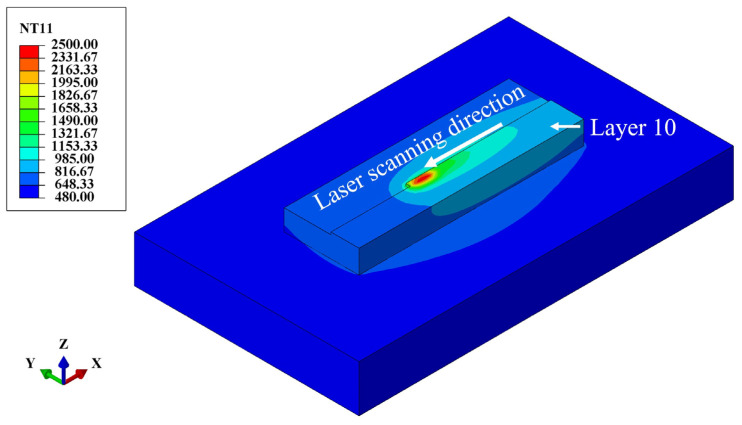
The temperature distribution during DED in layer 10.

Thermal analysis was used to predict the temperature distribution, heat flux, and cooling rate. This information was then imported into the mechanical analysis to predict material deformation and residual stresses. Figure 3 shows the temperature distribution during the deposition of layer 10. The maximum temperature of the melt pool is up to 2480 °C. It is ellipsoidal and moves in the scanning direction.

### 3.2. Model Validation

**Figure 4:**
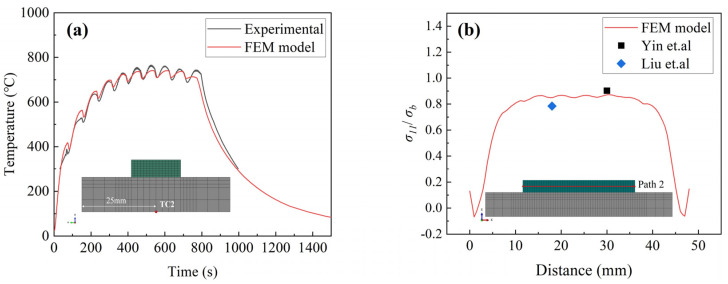
(**a**) Comparison of experimental and FEM-simulated TC2 point temperature history. (**b**) The normalized residual stress distributions (longitudinal residual stress/yield strength) along Path 2 [22,29].

During the critical process, the temperature of the center point of the substrate at point TC2 was measured using an infrared thermometer (SENTEST: NS10PH2SF). The thermal model was verified by comparing the measured temperature history with the predicted temperature history, as shown in Figure 4a. The predicted thermal history matches well with the experimental results. Both the predicted and experimental calculations show 15 peaks. The maximum difference in peak temperatures between the two is about 50 °C, which falls within the acceptable range. The distribution of normalized residual stresses along the central axis of symmetry (Path 2) of the deposited block is shown in Figure 4b. It can be seen that the predicted values obtained by the finite element model are comparable to the residual stress measurements of overlay welded Inconel 690 in the literature [29]. It can be seen that the model provides a reliable prediction of the temperature history and the residual stress distribution for the DED process.

### 3.3. Effect of Process Parameters on Temperature History

**Figure 5:**
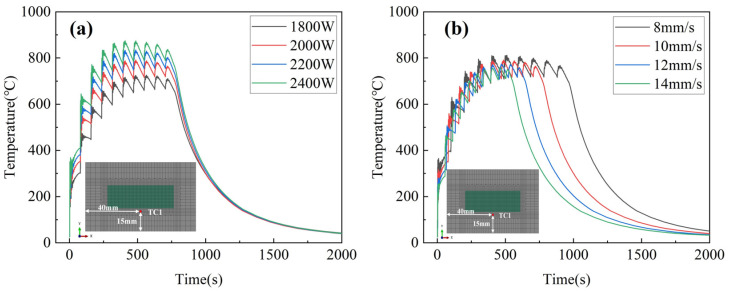

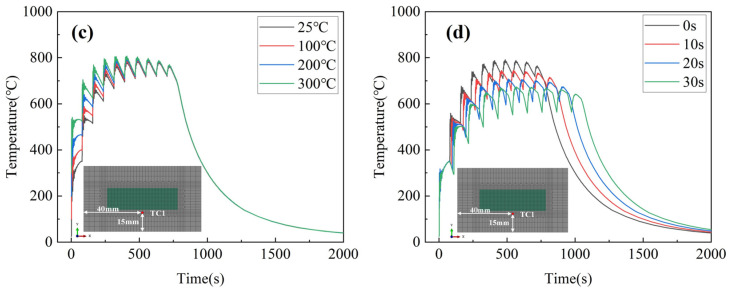
Influence of different process parameters on the temperature history at the TC1 point: (**a**) laser power; (**b**) scanning speed; (**c**) preheating temperature; and (**d**) interlayer dell time.

Figure 5 presents the results of the temperature field simulation in a room temperature environment. The temperature variation at TC1 under different process parameters was analyzed to understand the effect of the deposition process on thermal stress. The variables selected for the Inconel 690 directed energy deposition process include laser power, scanning speed, substrate preheating temperature, and interlayer dwell time, while other process parameters were kept constant. Figure 5a shows a significant increase in the peak temperature of each layer as the laser power increases. At a laser power of 2400 W, the peak temperature reaches its highest value of 874.6 °C during the sixth layer deposition, as depicted in Figure 5b. As the scanning speed increases, the peak temperature of each layer slightly decreases, and the heat dissipation time of each layer shortens. Figure 5c shows a significant increase in peak temperature for the first three layers as the substrate preheating temperature increases. The temperature history converges after the fifth layer of the deposition channel. As depicted in Figure 5d, the peak temperature of each layer decreases significantly with the increasing dwell time. At a dwell time of 0 s, the peak temperature is only 791.2 °C. At a dwell time of 30 s, the peak temperature is only 674.0 °C. To summarize, the peak temperature is significantly influenced by the laser power and dwell time, while the laser scanning speed has a minor impact. Additionally, the preheating temperature only affects the initial deposition of layers 1–4.

### 3.4. Effect of Process Parameters on Residual Stresses

**Figure 6:**
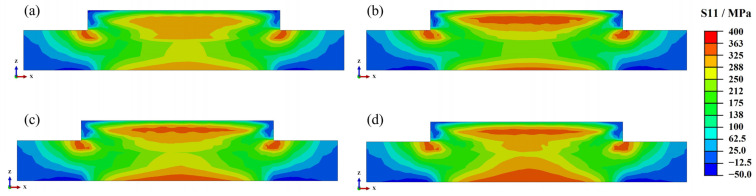
Residual stress distribution on the cross-section at different laser powers: (**a**) 1800 W; (**b**) 2000 W; (**c**) 2200 W; (**d**) 2400 W.

This section discusses the influence of laser power on residual stresses. The laser power directly affects the peak temperature, which, in turn, affects the residual stress. Our previous study indicates that longitudinal residual stress is the main stress component. Figure 6 shows the longitudinal residual stress distribution of Inconel 690 with different laser powers. The residual stress distribution of the parts processed with different laser powers is similar, with the residual stresses concentrated in the upper-middle part, the bottom of the substrate, and the left and right sides of the part bonded to the substrate. This concentration of stress makes these areas prone to structural buckling and fracture. Furthermore, the residual stresses’ magnitude increases as the laser power increases, and the area of high residual stresses (>325 MPa) expands. This law was also confirmed in our previous study [13].

**Figure 7:**
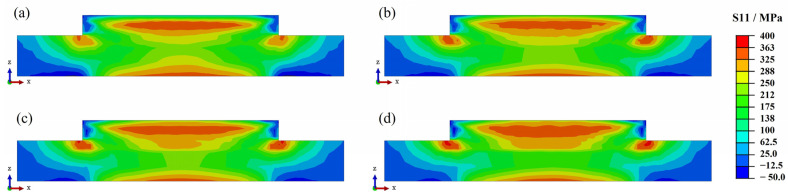
Residual stress distribution in the cross-section at different laser scanning speeds: (**a**) 8 mm/s; (**b**) 10 mm/s; (**c**) 12 mm/s; (**d**) 14 mm/s.

Figure 7 shows the longitudinal residual stress distribution of Inconel 690 at different scanning speeds. The residual stress distributions remain consistent across the four scanning speeds for each case, with only the high residual stress region (>325 MPa) expanding. In addition to the previous temperature analysis, it has been found that increasing the scanning speed has a minimal impact on both the peak temperature and temperature gradient. As a result, it has a limited effect on residual stresses. This finding is consistent with previous research on single-arm wall structures made of WAAM-molded Inconel 718 [30].

**Figure 8:**
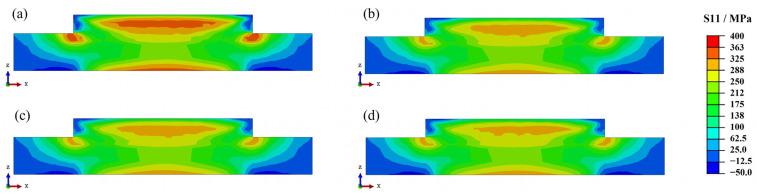
Residual stress distribution in the cross-section at different preheating temperatures: (**a**) 25 °C; (**b**) 100 °C; (**c**) 200 °C; (**d**) 300 °C.

The preheating temperatures for the welding process of Inconel 690 alloy were 25 °C, 100 °C, 200 °C, and 300 °C. Figure 8 shows the longitudinal residual stress distribution of Inconel 690 sections at different preheating temperatures. By increasing the preheating temperature to 300 °C, the residual stress distribution becomes similar to that at room temperature (25 °C). The stress level significantly decreases in the upper middle of the part and in the region of bonding with the substrate.

**Figure 9:**
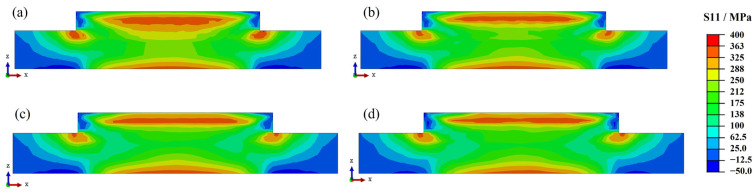
Residual stress distribution in the cross-section for different dwell times: (**a**) 0 s; (**b**) 10 s; (**c**) 20 s; (**d**) 30 s.

This study selected dwell times of 0 s, 10 s, 20 s, and 30 s based on the convergence of calculation and actual deposition efficiency. The longitudinal residual stress distribution of the cross-section under different dwell times is shown in Figure 9. The stress level decreases significantly in the upper and middle parts and the combined region with the substrate as the interlayer dwell time increases.

### 3.5. Mitigation Measures for Residual Stresses in DED

In additive manufacturing, the large temperature gradient between the moving melt pool and the deposited part results in a significant difference in thermal expansion, which leads to compressive stress. When this stress is above the yield point, plastic deformation occurs. As the heat source moves away and the region begins to cool, the material in the region contracts, resulting in residual tensile stress [31]. If the residual stress is above the tensile strength of the material, cracks will develop in the deposited part, severely inhibiting the life of the deposited part.

**Figure 10:**
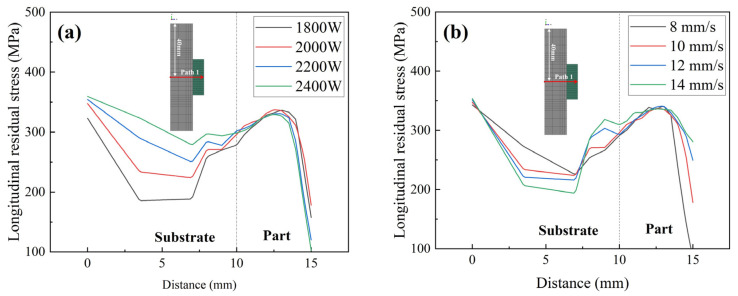

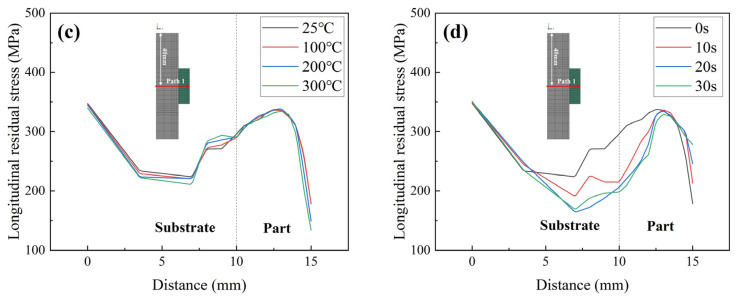
Residual stress in the YZ section along the normal direction. (**a**) Laser power; (**b**) scanning speed; (**c**) preheating temperature; and (**d**) interlayer dell time.

**Figure 11:**
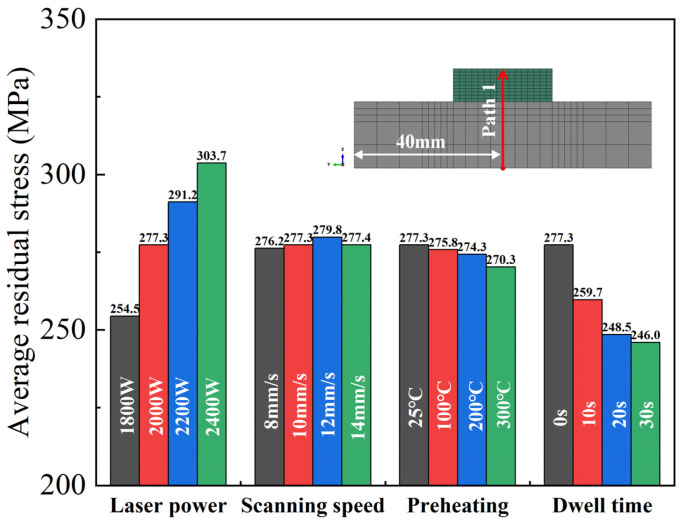
Comparison of longitudinal residual stress distribution along Path 1 with different process parameters. (**a**) laser power; (**b**) scanning speed; (**c**) preheating temperature; (**d**) Dwell time.

To clarify the influence of each process parameter on residual stress in the DED process, we selected the geometric center of the symmetry axis of the model as Path 1. The longitudinal residual stress distribution along the Path 1 direction is shown in Figure 10. Residual stresses in the deposited part increase initially, reaching a maximum at the 5th–6th deposited layer, and then continue to decrease. We calculated the average value of longitudinal residual stress along Path 1; the results are shown in Figure 11. The average level of longitudinal residual stresses increases with increasing laser power. This is because increasing the laser power leads to higher peak temperatures and cooling rates of the molten pool, as well as a corresponding increase in the temperature gradient, resulting in larger residual stresses. As shown in Figure 11, the residual stresses along the build direction are more likely to be influenced by the laser power rather than the scanning speed. This finding is also in line with the results of Mirkoohi et al. [32]. Longitudinal residual stresses in the build direction decrease with increasing substrate preheat temperature. The effect of preheating temperature on residual stresses is not as significant as previously reported in the literature [33]. This is because the model used in this study refers to a preheating temperature of 300 °C for actual Inconel 690 alloy welding, which is much lower than the preheating temperature of 700 °C used in the PBF process described in the literature. However, too high a preheat temperature can cause grain coarsening [34], which can affect the mechanical properties of the material. The inclusion of interlayer dwell time significantly reduces residual stresses. This is mainly attributed to the additional cooling that occurs between individual layers, which helps to reduce peak temperature and temperature gradient, which leads to a reduction in residual stress. This finding is supported by the existing literature [26]. In summary, to reduce residual stresses in components while ensuring specimen densification and molding efficiency, it is recommended to use a lower heat source power, a longer dwell time between layers, a higher preheating temperature, and an appropriate scanning speed.

## 4. Conclusions

This study established a finite element model of directed energy deposition molding of Inconel 690 alloy and investigated the influence of typical process parameters on temperature history and residual stress distribution. The main research conclusions are presented below.

(1) For the temperature field, the peak temperature of each layer during the molding process increases with the increase in laser power and preheating temperature, and decreases with the increase in scanning speed and interlayer dwell time. It was observed that substrate preheating has a significant effect only on the peak temperature of the first four layers.
(2) For the stress field, residual stress is mainly concentrated in the upper middle of the part, the bottom of the substrate, and the left and right sides of the bonding part with the substrate. The maximum value of the residual stress is close to the yield strength of Inconel 690 alloy. Residual stress increases with laser power and decreases with interlayer dwell time. Scanning speed and preheating temperature do not significantly affect residual stress.
(3) Lower heat source power, longer interlayer dwell time, higher preheating temperature, and appropriate scanning speed are beneficial for reducing residual stresses in Inconel 690 components, while ensuring specimen densification and molding efficiency.

Future studies could perform finite element modeling analysis on scanning strategies and complex components to accurately predict residual stresses and strains. This information can guide the additive manufacturing of real engineering components.

## Figures and Tables

**Table 1:** Temperature-dependent material properties of Inconel 690.

Temperature (°C)	Thermal Conductivity (W/m·°C)	Specific Heat (J/kg·°C)	Thermal Expansion Coefficient (10^−6^ 1/°C)	Young’s Modulus (GPa)	Poisson’s Ratio	Yield Strength (MPa)
25	11.92	0.43	12.50	208.27	0.31	387.13
100	13.10	0.45	12.88	203.45	0.31	335.96
200	14.67	0.47	13.38	196.80	0.32	301.58
300	16.23	0.49	13.88	189.92	0.32	282.67
400	17.78	0.50	14.38	182.79	0.32	270.81
500	19.34	0.52	14.89	175.43	0.32	262.70
600	20.88	0.54	15.39	167.82	0.33	256.81
700	22.43	0.56	15.90	159.97	0.33	252.36
800	23.98	0.58	16.41	151.88	0.33	248.87
900	25.52	0.61	16.94	143.51	0.34	194.31

## Data Availability

Data are contained within the article.
